# Navigating healthcare in higher education for students with gynaecological pain: an exploratory survey

**DOI:** 10.1186/s12905-025-03882-1

**Published:** 2025-07-05

**Authors:** Katie Paddock-Hall

**Affiliations:** Faculty of Health and Education, Manchester Metropolitan University, Brooks Building 53 Bonsall Street, Manchester, M15 6GX United Kingdom

**Keywords:** Womens health, Gynaecology, Healthcare services, Menstruation

## Abstract

**Background:**

University students face unique challenges in accessing healthcare, particularly women who experience painful gynaecological conditions. These conditions can impact their quality of life and educational engagement. Gynaecological pain is frequently misattributed as ‘normal’ dysmenorrhea, leading to delays in seeking medical advice and diagnosis. Research on how women with gynaecological conditions navigate healthcare is lacking in a UK higher education context. This study aims to explore university students’ experiences of gynaecological pain within higher education, and how they navigate healthcare.

**Methods:**

A web-based questionnaire integrating quantitative and qualitative items distributed to university students in England. The survey included the Menstrual Symptoms Index (MSI) to assess the severity and frequency of symptoms. Questions explored experience of accessing healthcare for gynaecological pain and the impact of symptoms on aspects of daily life and educational engagement. This project was supported by an advisory group comprising relevant stakeholders.

**Results:**

The study included 70 university students, predominantly aged 18–24. Gynaecological pain was highly prevalent, but 52% of participants had not spoken to a healthcare professional about this, 56% were not taking pain medication, and 80% had not sought university support. This was most typically due to normalisation of symptoms. Some participants had difficulties accessing healthcare around university commitments. Many reported negative experiences with healthcare professionals who dismissed or minimised their pain, particularly among younger participants. This pain impacted educational engagement with participants reporting difficulties in concentrating, attending classes, and completing assignments. Participants felt supported by individual university tutors but experienced difficulties navigating the procedural landscape of university to access accommodations. Students would benefit from greater awareness of the impact of gynaecological conditions among academic and healthcare professions.

**Conclusions:**

Gynaecological pain has a significant impact on university students’ educational engagement. There is a need for better support systems within universities, including improved awareness and understanding of gynaecological pain among staff and flexible academic policies. Training for healthcare providers to recognise and validate gynaecological pain is also essential. Addressing these issues can improve the quality of life for students and enhance their participation in educational settings.

## Background

Women can experience painful gynaecological conditions that impact their educational engagement. Gynaecological pain, originating from and around the female reproductive organs and pelvis, encompasses a range of conditions, including, dysmenorrhea (severe period pain), endometriosis, polycystic ovary syndrome (PCOS), fibroids, pelvic inflammatory disease (PID), and some cancers. Such conditions can impact quality of life, social and personal relationships, employment, educational engagement and attainment [1–12]. For many women, gynaecological pain and symptoms of associated conditions begin in adolescence or early adulthood, often coinciding with their time in further or higher education. Conditions linked to the menstrual cycle, such as dysmenorrhea and endometriosis, can result in pain during adolescence and young adulthood, with diagnoses of endometriosis typically occurring in late 20s or early 30s [13–15]. Other conditions, such as PCOS, PID, are not directly linked to the menstrual cycle, though symptoms of PCOS usually start around puberty, while PID most commonly affects sexually active women between the ages of 15 and 24 [16, 17].

Despite its prevalence, gynaecological pain is often misattributed as ‘normal’ by patients and healthcare practitioners (HCPs), particularly if it occurs around menstruation [1, 6, 18]. Consequently, many women do not seek medical advice [1, 12, 19, 20], and rely on low-quality online information to guide their health-seeking [19]. Evidence also suggests that many women have negative experiences of seeking healthcare support for gynaecological pain and associated symptoms. Patient-centred care entails working in partnership with patients and promoting medical agency to make autonomous, informed decisions about their health and healthcare, and is at the heart of many NHS and NICE guidelines [21–24]. However, many women experience “medical gaslighting”, where they feel their symptoms and concerns are dismissed by HCPs [19, 25–27]. This can further delay diagnosis of conditions that may have a complex and lengthy diagnostic process. For example, from symptom onset it takes on average between 7 and 9 years to diagnose endometriosis and about 4.3 years to diagnose PCOS [7, 14, 28, 29]. This is partly due to the non-specific nature of symptoms, like menstrual irregularities and pain, which can be mistaken for other conditions [7] and the limitations of current diagnostic tools [20, 30]. In secondary care in the UK, three-quarters of a million women are waiting for gynaecology treatment, not including those awaiting diagnostic tests or follow-up care [31, 32]. These delays in healthcare support or diagnosis can lead to worsening physical symptoms, impact quality of life, and cause psychological distress. For students, this can also disrupt educational engagement and achievement [6, 33].

Gynaecological pain significantly impacts women’s lives, particularly in education. Dysmenorrhea is a leading cause of school and work absenteeism [3, 12, 20], and chronic conditions such as endometriosis, can result in frequent absences, impaired concentration, reduced productivity, missing classes, poor exam performance, and lower academic achievement [2, 14, 34]. Although there is research on specific gynaecological conditions among university students and younger women [3, 12, 20, 35], there is a notable lack of studies focusing on the broader spectrum of gynaecological pain among university students. This gap is crucial because it overlooks the experiences of women who are early in their diagnostic journey or who have undiagnosed pain, and the unique experiences of navigating these issues and healthcare systems within higher education.

The transition to higher education presents unique challenges for university students. Students often need to register with new healthcare providers or navigate unfamiliar systems, and may struggle with self-advocacy in healthcare settings. Additionally, the effects of gynaecological pain can worsen academic pressures and social dynamics of university life, further impacting students’ wellbeing and educational engagement. Furthermore, the diagnostic process for many gynaecological conditions often involves multiple - sometimes invasive - procedures such as pelvic exams, transvaginal ultrasounds, and biopsies. Recovery times vary, with operative laparoscopy taking 3–8 weeks, and cervical biopsies up to a week [7, 30]. Scheduling these procedures and recovery periods around academic commitments poses additional challenges for women in education. Balancing academic responsibilities with health needs can be difficult, leading to delays in seeking care and worsening health [20].

This study aims to explore university students’ experiences of gynaecological pain within higher education, and how they navigate healthcare. Focus will include pain frequency, experience of symptoms associated with gynaecological conditions, perceived impact on educational engagement, and experiences of accessing healthcare for gynaecological pain and university support. There is limited research on experiences of healthcare in higher education, particularly regarding the broad spectrum of gynaecological pain. By understanding how students experience and navigate healthcare services and the impact on their educational engagement, we can improve the experience for women affected by these conditions and enhance their participation in educational settings.

## Methods

The study drew on a mixed methods approach comprising a survey that integrated quantitative and qualitative items. This provided a more comprehensive understanding of student experiences.

### Patient and public involvement

This project was supported by an advisory group comprising current university students with a history of gynaecological pain, education professionals including university disability coordinators, healthcare practitioners including primary care practitioners and practice nurses with a specialist interest in gynaecological health, and representatives of relevant charities and organisations. The advisory group informed the research question, survey design, and analysis.

### Sampling and recruitment

We employed a multi-faceted sampling and recruitment process to maximise reach and diversity in the sample. Locally, recruitment posters were distributed within local universities, approached student networks (including Black and Minority Ethnic, and women-focussed networks), and shared study information via internal email. Broadly, we promoted the survey via social media platforms (X, LinkedIn), contacted several relevant charities and organisations, and 159 Student Unions and Student Union representatives, however many had a policy to not promote research projects. Advisory group members also promoted the survey within their networks. Recruitment material included a link and QR code that directed participants to the online survey.

Ethical approval was obtained from the university’s ethics committee, and informed consent was collected from all participants.

Eligibility criteria:

- Assigned female at birth,
- Has experience of gynaecological pain, and.
- Is currently enrolled as a university student in England.

### Survey design

The survey was designed to explore university students’ experiences of gynaecological pain and how they access healthcare while studying. It consisted of five sections: 1) Participant Information and consent; 2) Experience of gynaecological pain and accessing healthcare during higher education; 3) demographic information; 4) consent for follow-up interview; 5) debrief with information on how to register with a General Practitioner (GP) and access medical care as a student. Section 2 included the Menstrual Symptoms Index (MSI) [36] - a tool used to assess the severity and frequency of 18 commonly reported menstrual symptoms, which are also linked to a range of gynaecological conditions. The MSI helps in understanding the impact of these symptoms on daily life. Qualitative data were collected via five items enquiring about participant experiences (1) speaking with HCPs or (2) university staff about their pain, and proposed changes to their (3) university or (4) healthcare experience, and (5) any further comments. Participants could also provide free-text responses to several items. This approach enabled a deeper understanding of the personal experiences and challenges faced by students. Participants could decline to respond to some survey items if they wished.

The survey was distributed online using a secure survey platform (Qualtrics).

### Data analysis

Survey data were entered into SPSS (V.29) and analysed using descriptive statistics. Analysis of free-text responses were broadly interpretive and informed by thematic reflective approaches [37, 38] to identify common patterns in students’ experiences and healthcare access. NVivo software (V.14) was used to assist with indexing and coding of qualitative data. KPH cross-referenced themes identified from the qualitative analysis with related data from the survey analysis.

## Results

### Participant demographics

Of 85 responses, 70 were included in the analysis. An overview of the sample is presented in Table 1. Most participants were aged 18–24, white, identified as female, and studied full-time at undergraduate level. There were similar proportions of local and commuter students, and those not currently in employment or working part-time. Most participants did not identify as being disabled or having a long-term impairment/illness.

**Table 1:** Participant demographics

Demographic category	Response	Frequency *n*(%)
Age	18–24	51 (73%)
	25–34	15 (21%)
	35–44	3 (4)%
	45–54	1 (1%)
	55–64	0 (0%)
	Older than 65	0 (0%)
Gender identity	Female	65 (94%)
	Transgender	3 (4%)
	Non-binary	2 (3%)
Ethnicity	African	4 (6%)
	Any other ethnic group	2 (3%)
	Any other Mixed or multiple ethnic background	4 (6%)
	Any other White background	4 (6%)
	Bangladeshi	1 (1%)
	English, Welsh, Scottish, Northern Irish or British	42 (60%)
	Irish	3 (4%)
	Pakistani	7 (10%)
	White and Asian	1 (1%)
	White and Black Caribbean	1 (1%)
Employment	Full-time employment	10 (14%)
	Not currently employed	19 (27%)
	Part-time employment	22 (31%)
	Zero hours contract	14 (20%)
	Other*	5 (7%)
Level of study	Undergraduate	45 (64%)
	Postgraduate	25 (36%)
Mode of study	Full-time	63 (89%)
	Part-time	7 (10%)
Status	Home	64 (91%)
	Overseas/International	6 (9%)
Local/commuter	Local	37 (54%)
	Commuter	30 (43%)
	Other**	1 (1%)
Health status	Disabled	7 (10%)
	Having a long-term impairment/illness	18 (28%
	None of the above	45 (64%)

* other forms such as “gig work”

**online learning

When asked if they had a diagnosis of a gynaecological and/or pelvic condition, 57% of participants declined to answer. Of those who did respond, 57% (17/30) had received a diagnosis, and 43% (13/30) had not. Disclosed diagnoses are presented in Table 2. Among participants with a diagnosed gynaecological/pelvic condition, four (24%, 4/17) did not identify as either disabled or having a long-term impairment/illness.

**Table 2:** Diagnoses

Condition	Frequency *n*(%)
Endometriosis	8 (11%)
Adenomyosis	1 (1%)
Polycystic Ovarian Syndrome (PCOS)	2 (3%)
Benign ovarian cysts	1 (1%)
Pelvic inflammatory disease	0
Dysmenorrhea	1 (1%)
Uterine fibroids	1 (1%)
Endometrial polyps	0
Gynaecological cancer	0
Prefer not to say	1 (1%)
Other*	5 (7%)

*Vaginal atrophy (*n* = 1); Vaginal/uterine atrophy (*n* = 1); precancerous cervical cells (*n* = 1); vaginismus (*n* = 1); no response (*n* = 1)

### Experience of gynaecological pain

Most participants typically experienced their pain on or around their period (62%, 41/66), whereas 38% (25/66) did not. 23% (12/52) typically experienced pain during or after sexual intercourse. Most reported experiencing pain for about a week each month (33%, 22/64) or a few days (29%, 19/64), which reflects the tendency for pain to occur around menstruation. Few participants experienced gynaecological pain most days (6%, 4/64) or every day (4%, 3/64). However, 27% (18/64) experienced pain for about two to three weeks each month. These findings not only highlight the variability in the frequency of gynaecological pain among participants, but that it is, nonetheless, frequent.

The most commonly reported symptoms from the Menstrual Symptoms Index (MSI) (Table 3) were tiredness/fatigue and mood changes/anxiety, each affecting 71% of participants. Poor concentration/memory affected 50%. Bloating/increased gas and stomach cramps were reported by 66%, joint pain/muscle cramps by 60%, lower back pain by 59%, and headaches/migraines by 47% - emphasising the widespread nature of pain-related symptoms. Despite this, most participants (56%, 37/66) were not taking medication for their pain, while 38% (25/66) were, and 6% (4/66) were unsure. Qualitative responses suggest that some participants managed their pain with over-the-counter painkillers; several were prescribed painkillers, anti-inflammatories, or antifibrinolytics. One participant was prescribed antidepressants due to the impact of the pain and are waiting to see a specialist as *“medications and painkillers [such] as codeine and paracetamol are no longer strong enough and I am not able to take anti-inflammatories”* P8.

**Table 3:** Menstrual symptoms index

Symptom	Frequency *n*(%)
Changes to/difficulties breathing	6 (7%)
Nausea/sickness/vomiting	30 (43%)
Constipation	22 (31%)
Dizziness/light-headiness/reduced co-ordination	31 (44%)
Poor concentration/memory	35 (50%)
Joint pain/muscle cramps	42 (60%)
Temperature fluctuations	27 (39%)
Disturbed sleep	30 (43%)
Diarrhoea	30 (43%)
Headaches/migraines	33 (47%)
Lower back pain	41 (59%)
Water retention	18 (26%)
Bloating/increased gas	46 (66%)
Stomach cramps	46 (66%)
Tiredness/fatigues	50 (71%)
Breast pain/tenderness	34 (49%)
Cravings/increased appetite	38 (54%)
Mood changes/anxiety	50 (71%)
Pain and/or burning, stinging, or itching of the urethra when urinating (Dysuria)	10 (14%)

### Impact of gynaecological pain

Results highlight the pervasive impact of gynaecological pain on various aspects of daily and university life, and underscore the need for improved support and resources to help manage this. Notably, 40% (25/63) felt their pain “almost always” interfered with their mood/mental health. Most felt that their pain sometimes affected their general physical activity (64%, 40/63), engagement in social activities (61%, 38/63), and their relations with other people (69%, 43/63). Over half reported that their pain sometimes impacted their employment (52%, 29/57) (Table 4).

Perceptions of the impact of their pain on their university experience were divided: 41% (29/64) felt it had an impact, 40% (28/64) felt it had not, and 10% (7/64) were unsure. Nonetheless, most participants reported a negative impact on a range of aspects of university life. For most, pain sometimes impacted concentration in lectures/seminars (65%, 38/59), attendance (56%, 33/60), and assignment completion (52%, 32/61).

**Table 4:** Impact on daily life

Impact Item	Frequency	*n* (%)
a. General physical activity	Almost always	13 (21%)
	Every so often/sometimes	40 (64%)
	Never	4 (7%)
	Rarely	6 (10%)
b. Mood/mental health	Almost always	25 (40%)
	Every so often/sometimes	35 (56%)
	Never	2 (4%)
	Rarely	1 (2%)
c. Relations with other people	Almost always	7 (12%)
	Every so often/sometimes	43 (69%)
	Never	4 (7%)
	Rarely	9 (15%)
d. Engaging in social activities	Almost always	14 (23%)
	Every so often/sometimes	38 (61%)
	Never	5 (8%)
	Rarely	6 (10%)
e. Sleep	Almost always	13 (21%)
	Every so often/sometimes	37 (60%)
	Never	7 (12%)
	Rarely	6 (10%)
f. Concentration in lectures/seminars	Almost always	11 (19%)
	Every so often/sometimes	38 (65%)
	Never	7 (12%)
	Rarely	3 (6%)
g. Attendance of lectures/seminars	Almost always	10 (17%)
	Every so often/sometimes	33 (56%)
	Never	11 (19%)
	Rarely	6 (10%)
h. Completing assignments	Almost always	9 (15%)
	Every so often/sometimes	32 (52%)
	Never	10 (17%)
	Rarely	11 (18%)
i. The physical aspects of my degree programme	Almost always	6 (21%)
	Every so often/sometimes	10 (36%)
	Never	9 (31%)
	Rarely	4 (14%)
j. Commuting to university	Almost always	9 (16%)
	Every so often/sometimes	26 (46%)
	Never	13 (23%)
	Rarely	9 (16%)
k. Employment	Almost always	6 (11%)
	Every so often/sometimes	29 (52%)
	Never	9 (16%)
	Rarely	13 (23%)

### Healthcare access

All respondents (100% 64/64) were registered with a GP. For students who moved between term-time and home addresses, 61% (19/31) were not registered as temporary patients, 29% (9/31) were, and 10% (2/31) unsure. Participants were either unaware they could register as temporary patient (*n* = 4), did not know how (*n* = 3), or did not feel the need to use GP services while studying (*n* = 3).

Over half of participants (52%, 34/66) had not spoken to a healthcare professional (HCP) about their gynaecological pain, whereas 43% (30/66) had, and 3% (2/66) were unsure. The most common reasons for not speaking to a GP were that respondents did not consider it a big enough issue to speak to a GP about it (41%, 14/34), it is normal to experience that type of pain (35%, 12/34), and that the pain is not so bad that they feel they need to see a GP about it (32%, 11/34) (Table 5). Qualitative responses suggest a lack of confidence that HCPs would find solutions: *“I did not think there was anything they could do”* P50.

**Table 5:** Reasons for not speaking with GP

Reasons	Frequency *n*(%)
It is normal to experience this type of pain	12 (35%)
The pain is not so bad that I feel I need to see my GP about it	11 (32%)
I do not think this it is big enough of an issue to see the GP about it	14 (41%)
I do not feel comfortable discussing this issue with my GP (whether female or male)	1 (3%)
I cannot get an appointment	4 (12%)
I am not registered with a GP	1 (3%)
Other: Please specify	5 (15%)

Many (38%, 22/58) were dissatisfied with their current access to healthcare for gynaecological pain/conditions, 29% (17/58) of were satisfied, and 33% (19/58) unsure. Most respondents were not confident that they could access healthcare for their gynaecological issues when they needed it (43%, 25/58), but were confident they would be referred to appropriate services (47%, 27/58). However, 60% (35/58) doubted timely referrals, and only 22% (21/58) were confident in this. Qualitative data suggested that many participants were still waiting for referrals and *“continuing to fight to be heard by medical professionals”* (P30) whilst managing their symptoms. Others did not pursue further support after initial disappointment with HCPs. Notably, a large proportion of participants found it difficult to schedule healthcare appointments around university commitments (45%, 26/58), and to schedule GP appointments when needed (48%, 28/58):

*“due to attending university and working every other free day i do not have time to book an appointment”* P47.

It was difficult to plan university obligations and employment while waiting for a GP appointment/phone call, or referral. Healthcare would be more accessible with changes to appointment-booking systems, allowing patients to book online instead of relying on calling the GP surgery at 8 a.m., and for follow-up telephone appointments at a specified time and “not general large windows for phone appointments” (P36).

*“…not knowing when your next appointment is going to be is quite concerning and makes it difficult to plan around University and my studies.”* P30.

Some also suggested a reduction in prescription charges for students. There were mixed perceptions of access to medication for and information about their gynaecological pain/condition. While 45% (26/58) felt they had access to necessary medication *and* information, 36% (21/58) felt they lacked sufficient information, and 22% (13/58) felt they did not have access to medication when needed.

### Healthcare experiences

Students’ experiences with healthcare services are largely consistent with existing literature, with negative experiences with HCPs, feelings of dismissal, and needing multiple GP appointments to get support. Participants citied difficulties coping with lengthy waiting lists and referrals, and little follow-up following a diagnosis and/or medical procedure, feeling that HCPs *“…just throw out a diagnosis and send me out the door…”* (P37).

Many participants described being *“gaslit”* and *“dismissed”* about their symptoms by HCPs who *“did not take it serious”* (P97). Including participants who later received a diagnosis of a gynaecological condition. This dismissal resulted in significant delays to receiving medical support for their symptoms, and/or a diagnosis. Participants felt that experiences would be improved if HCPs took patients’ pain severity and concerns seriously, without normalising symptoms, *“less dismissal of pain as ‘just that time of the month’”* (P14), and *“not just brush it off as a non issue”* (P25).

Conversely, some participants felt listened to and supported by HCPs, or felt their experience was positive because they eventually received a diagnosis and/or medication for their symptoms. However, some experienced a range of symptoms, which may have helped to convey the impact of their pain:

*“I can’t help but wonder if I hadn’t have had visible symptoms (I.e*,*. Being sick*,* then my pain might not have been taken as seriously).*” P57.

Some participants’ diagnostic journeys were interrupted by misdiagnosis, symptoms attributed to their other conditions or issues such as *“university pressure…”* (P23), “stress” (P9), or “*my periods being so bad was my own fault as I was overweight”* (P8). Others highlighted that though some of their symptoms were being treated, they continued to manage others on their own:

*“… they changed my pill and stopped irregular bleeding but…I am still dealing with pain”* P21.

Participants’ age influenced their healthcare experiences. Some reported difficulties agreeing with HCPs on suitable next steps to achieve a possible diagnosis, often because they were young:

“…*gynaecologist told me I wouldn’t have anything because I was young”* (P18).

Others were considered too young to warrant further medical exploration, such as diagnostic laparoscopy:

*“…my GP was quite dismissive and told me there was no point pursuing surgery for a diagnosis of endometriosis given my age”* (P43).

Participants also reported difficulties agreeing on treatment options, and felt pressured to engage in treatments they did not want, often due to their age and hesitations around hormonal contraception. For instance, P22 was not sexually active so was denied an IUD and encouraged to take hormonal contraception:

*… i’m not sexually active they wouldn’t typically give this [IUD] to me and wanted me to take the pill (something i didn’t want to do)…*.

Relatedly, P55 identified as transgender, and felt that HCPs dismissed their feelings regarding proposed treatment options, to prioritise their fertility:

*“…everyone has been so dismissive of my thoughts and feelings*,* keep trying to pressure me to take the pill*,* and are ignoring the extra level of distress i get around this being trans masc. and focusing on saving my fertility as (and i quote) ‘you are a woman of childbearing age so we would never consider that’…”* P55.

### Support from university

Participants were asked if they had spoken with university staff (such as tutors) about their health issues or engaged with university support systems. Despite many experiencing some impact on their university commitments, 80% (51/64) had not spoken with staff, 19% had (12/64), and 2% were unsure (1/64). Though some participants identified has having a disability (10%, 7/70), or long-term impairment/illness (28%, 18/70), just 29% (20/70) of these had informed their university’s disability and inclusion service of their condition(s). The main reasons for not speaking to university staff (such as a tutor) about gynaecological pain were feeling the pain’s impact was not significant (46%, 24/54), believing staff did not need to know (44%, 23/54), discomfort discussing the issue (regardless of gender) (24%, 13/54), and embarrassment (20%, 11/54):

*“can’t imagine feeling 100% comfortable speaking to my dissertation supervisor (a man) for example about my reasons for needing to cancel a meeting or postpone a task because of my pain.”* P43.

Others, (19%, 10/54) did not know who to speak with at university, or felt it was normal to experience gynaecological pain (19%, 10/54).

Those who had liaised with university staff about their pain tended to report positive experiences with *“super supportiv*e” (P15) and *“really understanding*” (P55) staff, including personal tutors and module lecturers. Participants felt their circumstances were understood, and found comfort in being able to tell someone at the university. They benefited from assessment extensions or deferrals, and from tutors accepting occasional absences.

*“I spoke to my lecturers about my condition when I couldn’t attend classes due to the pain and they were all supportive. It was a positive experience as I didn’t feel judged and was able to focus on my recovery.”* P46.

However, some participants who sought accommodations, such as assignment extensions for mitigating circumstances, encountered difficulties navigating central university procedures with the administrative burden of repeatedly requiring “*proof of your illness when you are at your worst”* (P8). This became more complex and burdensome for those who needed multiple instances of accommodations for the same or related reasons. Additionally, some conditions require ongoing treatment and/or invasive procedures, which can involve a recovery period that will further impact university engagement. Participants highlighted that students would benefit from the support of staff who understand the physical and mental impact of this, and of “*the impact that pain and waiting for surgery has on students and that we cannot just bounce back once that surgery happens*,* especially when a recurring illness such as endometriosis is involved”* (P8). Consequently, they emphasised the need for flexible and accommodating internal policies regarding attendance and extension/submission, to minimise the negative impact on educational engagement “…*so that we can submit our best work”* (P24).

Similarly, students would benefit from flexible lecture delivery such as hybrid or *“streamed lectures for when my pain is to bad to attend in person so i don’t fall behind”* (P22). Scheduling sessions to reduce travel between buildings or campuses would also lessen discomfort:

*“…I get lower back and upper leg pain with cramps and it makes walking uncomfortable*,* especially when it’s cold. At the moment every teaching session for a single module can be held in a different space all across campus so it’s unpredictable.”* P2.

Participants highlighted the importance of raising awareness among all university staff about the impact of gynaecological pain and related conditions, and for “…*academic staff to recognise the impact that chronic illness/pain can have on students’ studies”* (P22). This would help to foster a better understanding of the challenges faced by students and how staff can support them, and to minimise disruption to their education.

## Discussion

Little research has explored the unique experiences of navigating healthcare services and higher education systems for university students with painful gynaecological conditions. Such conditions are highly prevalent among this population due to the typical onset of symptoms around adolescence and early adulthood, regardless of links to menstruation [13–15]. This study highlights the impact of gynaecological pain on university students’ academic and personal lives, and the complexities of accessing healthcare while studying. This includes navigating the procedural landscape within higher education to access reasonable adjustments amidst normalised pain and medical gaslighting.

Despite the frequency of pain and symptoms that interfere with educational engagement and daily activities, many participants did not seek medical or university support. This reluctance was often due to the sensitive and personal nature of gynaecological issues [20], and also rooted in internalised normalisation of gynaecological pain with the belief that symptoms are not “bad enough” to warrant disclosure or support. Whilst this is a common barrier to help-seeking [18], it has not previously been identified in the context of higher education. Furthermore, consistent with findings in the growing body of literature on disability and chronic illness in higher education, many participants were either unaware of their eligibility for support, or chose not to engage with university services due to fear of stigma or complex institutional procedures [39–46]. Thus, findings suggest that initial barriers to health-seeking for students with gynaecological pain relate to the complexity of identifying that an issue warrants medical input, feeling legitimate in seeking medical and/or institutional support, then knowing how to achieve this within health and university settings.

Findings reiterate the prevalence of ‘medical gaslighting’ and suggest inconsistencies in the provision of patient-centred care, leading to delays in diagnosis or treatment [26, 47, 48], and exacerbating the impact on students’ lives and educational engagement. Though hormonal contraception is a typical method of symptom-relief for PCOS, endometriosis, and managing irregular periods [49–51], common concerns around hormones, side-effect anxiety, a desire for ‘natural’ menstruation [52] were minimised by HCPs. Findings also uniquely highlight how patient age can influence healthcare experiences, with some participants feeling pressured to engage in treatment options that prioritise fertility, undermining their medical agency [53], and not just for participants with endometriosis [54]. Fertility preservation is a key consideration in NICE guidance for women of reproductive age [55], as fertility can be compromised by conditions such as endometriosis, PCOS, and gynaecological cancers, and treatments such as surgery, chemotherapy, and radiotherapy [10, 55–60]. As per NICE guidance, patients should be offered information about the potential impact of their condition or treatment options on their fertility [49, 55, 61], but guidance also emphasises shared decision-making as part of patient-centred care [61, 62]. Discussions should enable patients to feel their priorities and concerns have been considered. However, further research is needed on the experiences of transgender and nonbinary people in this context as they face alternative barriers and discriminatory or invalidating experiences [34, 63–65].

Further findings suggest tension between students’ medical agency and university policies. Students with chronic illnesses often attempt to maintain a sense of control over their bodies and academic responsibilities through health routines and strategies [53]; this includes managing symptoms in the comfort of a home environment when experiencing gynaecological pain [20]. However, inflexible institutional structures and policies undermine this agency through strict attendance monitoring, limited opportunities for online or recorded lectures, and the administrative burden of proving illness to access accommodations. This becomes more complex and burdensome for those who need multiple instances of support for the same or related reasons, and staff may not understand the fluctuating nature of chronic or cyclical conditions [48]. Such institutional practices often fail to adequately support chronically ill students, and can lead to feelings of alienation and mistrust of institutional services, further impacting educational engagement [42, 66]. Nonetheless, participants benefitted from supportive and accommodating university staff, or “academic allies” [42], who understood their circumstances and authorised assignment extensions or absences. These experiences helped to facilitate educational engagement in a way that reinforces students’ sense of agency [42, 66]. However, reliance on individual staff for support underscores the need for flexible academic policies and practices that reduce barriers to accommodations. This includes reducing the administrative burden proof of illness policies for chronic or recurring conditions.

Educational strategies are needed to foster person-centred healthcare and university services. Participants emphasised the need for greater awareness of gynaecological conditions among university staff and– in particular - HCPs, with improved communication strategies in primary and secondary care to identify potential treatment/support options through shared decision-making. Greater access to symptom support whilst patients await referrals can also minimise long-term symptom impact [32, 67], and help them feel listened to.

The Women’s Health Strategy for England (WHS) [68] and The Primary Care Recovery Plan (PCRP) [69, 70] aim to address key gaps in women’s health experiences. This includes improving HCP training on women’s health, reducing systemic barriers such as the ‘8am scramble’ for GP appointments and long waits for gynaecological referrals, by enhancing digital and out-of-hours services and coordinated care through Women’s Health Hubs (WHH) [32, 68–71]. Such challenges are particularly acute for students navigating university commitments, and moving location during their studies; further research is needed on the efficacy and implementation of WHHs, and whether these also benefit women in higher education. Improved digital healthcare and streamlined options for access across ‘home’ and ‘university’ residencies, could also minimise disruption to educational engagement.

The WHS also aims to integrate menstrual and gynaecological health into statutory education guidance [72], which could improve awareness of gynaecological issues and encourage women’s medical agency. However, schools have flexibility in how they deliver this content within their curriculum, which may lead to inconsistent information quality and outcomes, warranting further research [72], and will not benefit women currently at the end of compulsory education. Findings from the present study suggest that women in higher education would benefit from targeted outreach programmes to promote earlier health-seeking and medical agency, particularly as this population may just start experiencing gynaecological pain and symptoms of associated conditions, and navigating healthcare services. Campus-based health promotion events could improve awareness for staff and students, with positive outcomes identified for initiatives for physical activity and mental health [73–75]. Akin to statutory guidance for compulsory education, any such interventions should be designed in collaboration with relevant stakeholders and experts.

Although the current survey is the first to explore the perceived impact of university students’ experiences of gynaecological pain within higher education, and how they navigate healthcare, there are some limitations. Recruitment was impacted by university policies to not promote research projects and thus the generalisability and diversity of the sample is restricted. Despite the modest sample, the data yield results to inform future larger-scale studies. There are inherent limitations in self-reported data including potential recall and social desirability responses, though self-report surveys remain a widely used practical method for collecting subjective experiences. There is the possibility of selection bias where participants who experienced more severe symptoms or negative experiences were more motivated to participate, which is a common challenge in such research. Future research should aim to include a larger and more diverse sample to ensure the findings are relevant to the broader higher education population.

## Conclusion

Gynaecological pain significantly affects university students’ educational engagement and quality of life. University policies and practices must be flexible to accommodate students who experience gynaecological pain and symptoms of associated conditions, including those without a formal diagnosis. Barriers to accessing healthcare and negative healthcare experiences continue to contribute to the underreporting and undertreatment of gynaecological conditions. These issues may be minimised with the implementation of the Women’s Health Strategy and The Primary Care Recovery Plan, but further evaluation is needed to determine their impact. Nonetheless, university students would benefit from a targeted approach to improve understanding of gynaecological health and navigating healthcare services around university commitments, and training healthcare providers to validate experiences of gynaecological pain. By addressing these issues, we can improve the quality of life for students and enhance their participation in educational settings.

## Data Availability

The datasets analysed during the current study are not publicly available but are available from the corresponding author on reasonable request.
